# Targeting the Perialar Space for Enhanced Nasolabial Fold Rejuvenation: A Cadaveric Study

**DOI:** 10.1111/jocd.70698

**Published:** 2026-02-01

**Authors:** Song Eun Yoon, Jung‐Woo Choi, Yong‐Seok Nam, In‐Beom Kim

**Affiliations:** ^1^ Department of Anatomy, Catholic Institute for Applied Anatomy, College of Medicine The Catholic University of Korea Seoul Republic of Korea; ^2^ Department of Anatomy, School of Medicine Kyungpook National University Daegu Republic of Korea

**Keywords:** deep pyriform space, dermal fillers, nasolabial fold, perialar space, volumization technique

## Abstract

**Background:**

Nasolabial folds, or “smile lines,” result from the interaction of several anatomical structures, including the levator labii superioris alaeque nasi and the zygomaticus major and minor muscles, which place continuous tension on the dermis. Although the deep pyriform space is a common target for treating these folds, filler migration from this space can cause undesirable outcomes.

**Aims:**

To examine the anatomical characteristics and potential benefits of using a newly identified medial space: the perialar space, adjacent to the deep pyriform space, for nasolabial fold volumization to facilitate more effective and stable filler placement.

**Methods:**

Twenty‐two fresh cadavers were used (44 hemifaces: 13 male, 9 female; mean age 81.98 years) without trauma, surgery, or major deformities. Dyed injectate was administered into the deep pyriform and perialar spaces. Cannula resistance during insertion was measured, and injectate distribution was assessed through comprehensive dissection.

**Results:**

The mean forces at the first and second puncture sites were 1.70 ± 0.58 N and 3.29 ± 1.35 N, respectively. Dissection confirmed injectate placement in a space adjacent to, but distinct from, the deep pyriform space. Statistical analysis showed significant differences in tissue resistance between sites (*p* < 0.05). The perialar space thus represents a useful alternative for nasolabial fold volumization. Histological evaluation confirmed distinct boundaries, supporting its suitability as a stable and reliable site for filler injection.

**Conclusions:**

This study shows the potential of the perialar space for enhancing aesthetic outcomes; reducing complications and filler migration; and achieving more stable enhancements in facial rejuvenation procedures than existing techniques.

## 1 Introduction

Nasolabial folds, commonly referred to as “smile lines,” are skin creases that extend from the side of the nose to the corners of the mouth, accentuating facial expressions [1]. Deepening of the nasolabial folds is attributed to multiple anatomical and physiological factors. These folds are primarily affected by the levator labii superioris alaeque nasi (LLSAN), the zygomaticus major, and the zygomaticus minor muscles, which exert continuous tension on the dermis during facial movement. Other contributors include additional muscular activity that generates continuous tension on the dermis, subcutaneous fat volume deficiency, nasolabial fat pad sagging, and skin laxity associated with aging [1, 2, 3, 4, 5]. Collectively, these elements cause the formation and deepening of these folds, highlighting the complex interaction among muscular dynamics, soft tissue support, and age‐related structural changes.

Various treatment methods have been employed to mitigate the deepening of nasolabial folds, with the widespread use of cosmetic dermal fillers injected into targeted areas such as the Ristow and the deep pyriform space. This addresses the root causes by augmenting volume and providing structural support [6, 7]. However, complications such as filler migration have occasionally been reported, leading to undesirable outcomes in patients [8, 9, 10, 11, 12]. These challenges underscore the need for better volumization techniques that are effective and safe. Therefore, the aim of this study was to present a novel approach targeting a space adjacent to the medial border of the deep pyriform space and the medial border of the LLSAN, which could provide a viable alternative to traditional methods.

## 2 Materials and Methods

### 2.1 Participants and Specimen Preparation

In this study, we examined 22 fresh cadavers (44 hemifaces: 13 male and 9 female individuals), with a mean age of 81.98 (range, 58.7–103.7) years (Table 1). Written informed consent was obtained from the patient when he/she was alive and from the next of kin when the patient passed away. Cadavers with signs of trauma, surgery, or significant deformities were excluded. Warm water and gelatin powder (Duksan Pure Chemicals. Co. Ltd., Ansan, South Korea) were mixed at a 10:1 ratio as the injection reagent, and cobalt blue or red ink (Alpha Colors Co., Seoul, South Korea) was added to facilitate comparison of the spaces. Following the Korean Act on the Anatomical Dissection and Preservation of Corpses as amended, this study was performed after review and receiving ethical approval from the Institutional Review Board of the College of Medicine, The Catholic University of Korea (approval number: MC21EISI0103).

**TABLE 1:** Average age of male, female, and combined cadavers.

	Male (*n*)	Female (*n*)	Total (*n*)
Age, years	79.19 ± 11.1 (13) [58.7–103.7]	86.01 ± 7.52 (9) [74.6–99.3]	81.98 ± 11.04 (22) [58.7–103.7]

### 2.2 Injection Protocol

**FIGURE 1:**
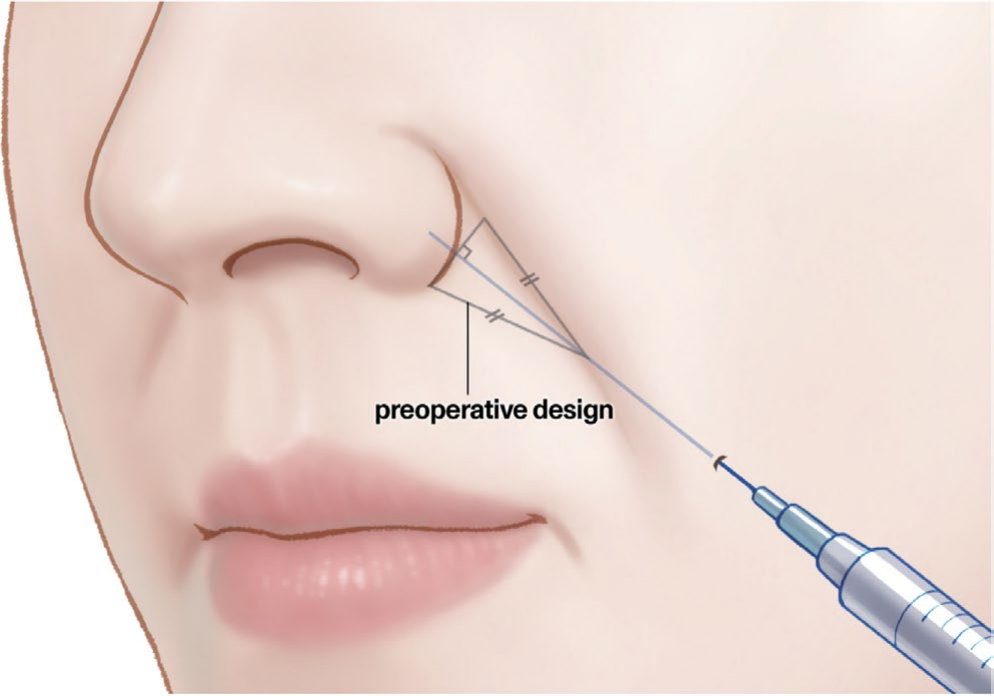
Schematic illustration of the filler injection procedure targeting the nasolabial fold using an imaginary isosceles triangle.

Prior to nasolabial fold injection, the targeted region was visualized as an isosceles triangle. A baseline was established from where the fold intersected the nasal alar to its inferior margin. The midpoint of this baseline was identified, and a line was drawn perpendicular to the fold. The intersection point of this line was located at the nasolabial fold and the ideal puncture site was identified approximately 1 cm along this line from the intersection point (Figure 1). The cannula was maneuvered within this triangle during the injection procedure. An initial skin perforation was created using a 21‐gauge needle and a 23‐gauge; a 50‐mm 1 cannula, connected to a Nextech DFS500 force gauge (Nextech, Bangkok, Thailand), was inserted through the puncture hole. As the cannula advanced, the initial fascial tension was measured to identify the first significant resistance point. Upon penetration of this layer and verification of the correct placement of the cannula on top of the periosteum within the deep pyriform space, the force gauge was detached, and a syringe preloaded with dyed injectate was connected to deliver 0.5–1 cc injectate in a retrograde fashion. The cannula was subsequently reattached to the force gauge and advanced toward a deeper target site adjacent to the medial border of the LLSAN. The force required to breach this second fascial layer was measured at the second tension point, where increased resistance was experienced. Following penetration, the force gauge was removed and a syringe containing a different colored gelatin injectate was attached for the second 0.5–1 cc injection.

### 2.3 Post‐Injection Procedure and Analysis

After injection, the cadavers were refrigerated to preserve the filler's shape for subsequent dissection. Detailed dissections were performed to assess the distribution and integration of the injectate within the facial tissues. First, an incision was made along the skin crease between the nose and eyes to expose the underlying structures. The skin and soft tissues were carefully removed to identify the lower part of the orbicularis oculi (Oo), levator labii superioris (LLS), and LLSAN. In the superficial layer, the Oo was first identified in the orbital region, followed by the zygomaticus minor and LLSAN, extending inferiorly. In the deeper layers, the LLS and transverse nasalis muscle were identified. The LLS was transected perpendicular to its fiber direction at the middle of its belly, and a part of the LLSAN was excised to expose the dyed injectate beneath the muscles near the nose. The distribution of injectates within the site was assessed. One cadaveric specimen was used for histological evaluation. The tissue was cut into sections of approximately 7 μm thickness and stained using standard hematoxylin and eosin (H&E) and Masson trichome.

Statistical analysis was performed using the Statistical Product and Service Solutions software (SPSS version 24.0; IBM, Armonk, NY, USA). The left and right sides and the 1st and 2nd puncture sites were compared using a paired *t*‐test. Results with a *p*‐value < 0.05 were considered statistically significant.

## 3 Results

**FIGURE 2:**
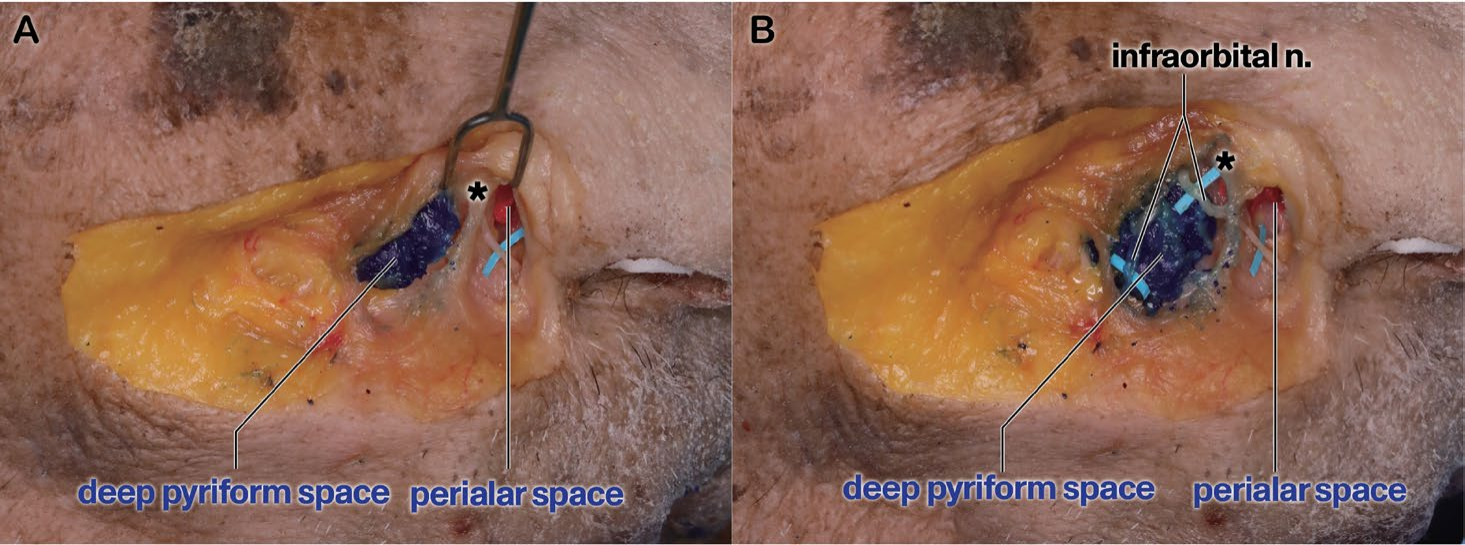
Cadaveric dissection showing the deep pyriform space and the perialar space after filler injection. (A) The deep pyriform and perialar spaces are clearly separated by fascial tissue (indicated with an asterisk). The asterisk represents the fascial boundary separating the two spaces. (B) Complete dissection demonstrating that the dyed filler has been injected beneath the infraorbital nerve.

As shown in Figure 2, two spaces are distinctly separated, with clearly defined borders. The space filled with blue injectate represents the deep pyriform space located lateral to the LLSAN and beneath the LLS. The red injectate indicates a newly identified space located medial to the deep pyriform space. This new space is defined laterally by the LLS, medially by the alar fibrofatty tissue, and superiorly by the roof formed by the transverse nasalis muscle. The infraorbital nerve runs above the injectate in the deep pyriform space (Figure 2B).

The mean force (±standard deviation) at each puncture site was measured using a force gauge, and the results are presented in Table 2. The mean force at the first puncture site was 1.70 ± 0.58 N, whereas that at the second site was 3.29 ± 1.35 N. A significant difference was observed between the sites (*p* < 0.05). Furthermore, the mean force at the first puncture site was 1.49 ± 0.44 N on the right side and 1.92 ± 0.64 N on the left. For the second puncture site, the mean force was 3.00 ± 1.51 N on the right and 3.59 ± 1.11 N on the left. Significant differences between the right and left sides were noted at both puncture sites. No significant differences were observed between the male and female cadavers.

**TABLE 2:** Comparative analysis of tension measurements at the first and second puncture sites.

	$\text{Unit}:N;\text{mean}\pm \mathrm{SD}\;\left(N\right)$		
	Male (*n*)	Female (*n*)	Total
1st puncture			
Right	1.51 ± 0.46 (13) [0.90–2.70]	1.46 ± 0.42 (8) [0.80–2.30]	1.49 ± 0.44 (21)** [0.80–2.70]
Left	2.05 ± 0.61 (13) [1.20–3.40]	1.69 ± 0.69 (7) [0.70–2.80]	1.92 ± 0.64 (20)** [0.70–3.40]
Total	1.78 ± 0.60 (26) [0.90–3.40]	1.57 ± 0.55 (15) [0.70–2.80]	1.70 ± 0.58 (41)* [0.70–3.40]
2nd puncture			
Right	3.03 ± 1.56 (13) [0.80–5.60]	2.95 ± 1.54 (8) [0.80–5.90]	3.00 ± 1.51 (21)** [0.80–5.90]
Left	3.79 ± 1.04 (13) [2.30–5.70]	3.21 ± 1.20 (7) [1.40–4.70]	3.59 ± 1.11 (20)** [1.40–5.70]
Total	3.41 ± 1.36 (26) [0.80–5.70]	3.07 ± 1.35 (15) [0.80–5.90]	3.29 ± 1.35 (41)* [0.80–5.90]

* 1st vs. 2nd punctures: *p <* 0.05.

** Right vs. left sides at the 1st and 2nd puncture sites: *p <* 0.05.

**FIGURE 3:**
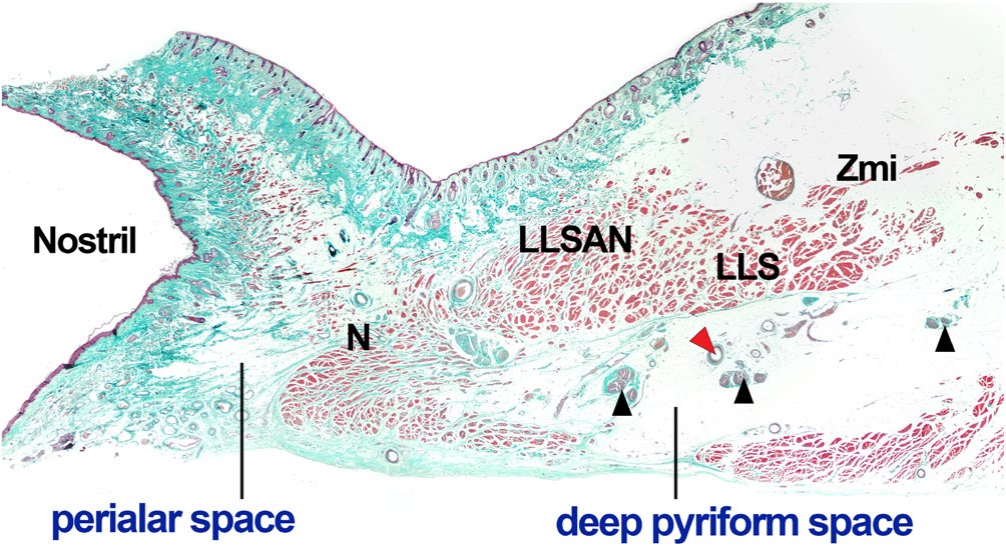
Histological section demonstrating the spatial arrangement of the perialar space and deep pyriform space. The N, LLSAN, LLS, and Zmi muscles are observed, with the neurovascular structures indicated with arrowheads. LLS, levator labii superioris muscle; LLSAN, levator labii superioris alaeque nasi muscle; N, nasalis muscle; Zmi, zygomaticus minor muscle.

The histological analysis further demonstrated clear anatomical boundaries separating the perialar and deep pyriform spaces (Figure 3). The histological sections revealed dense fibrous connective tissue and the transverse nasalis muscle surrounding the space.

## 4 Discussion

Since its first introduction as a potential space for volumization, the deep pyriform space has been widely used for filler injections. This midface cavity is bound medially by the depressor septi nasi and laterally by the deep medial cheek fat and lip elevator [6]. Injection into this space has been highly effective in diminishing the nasolabial folds, leading many practitioners to adopt it as an ideal injection site.

**FIGURE 4:**
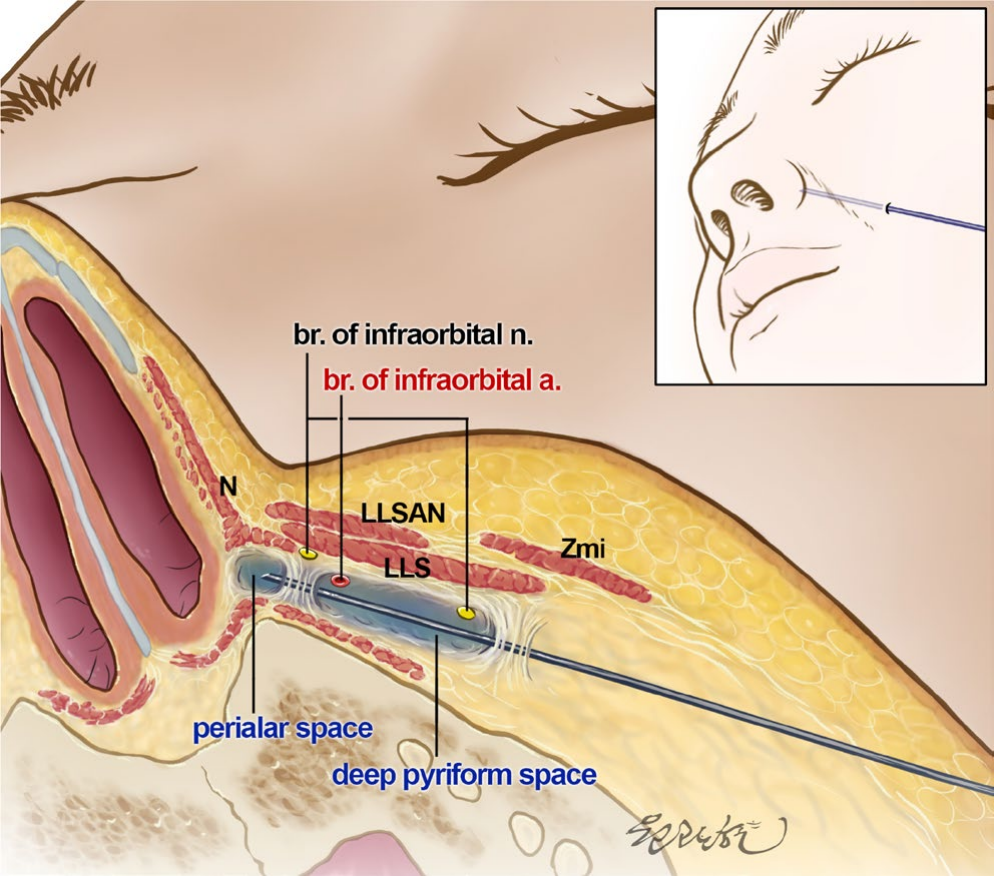
Schematic illustration of the anatomical boundaries in nasolabial fold volumization. This illustration shows the anatomical separation between the deep pyriform space and perialar space. LLS, levator labii superioris muscle; LLSAN, levator labii superioris alaeque nasi muscle; N, nasalis muscle; Zmi, zygomaticus minor muscle.

As shown in Figure 2, the medial space, the perialar space, is distinctly separated from the deep pyriform space. Accessing this newly identified space requires that practitioners guide the cannula more medially than when targeting the deep pyriform space, resulting in a sensation of increased tension. This increased tension is evident as the cannula penetrates not only the fascia of the LLS but also the LLSAN and the transverse nasalis muscle. Consequently, as illustrated in Table 2, the tension measured at the second puncture site is considerably higher than that measured at the first puncture site (3.29 ± 1.35 N vs. 1.70 ± 0.58 N). The first puncture primarily encounters the lateral border facial support provided by the LLS, whereas the second penetrates a more complex structure defined by the fascial tissues of the medial border of the LLS, LLSAN, and transverse nasalis muscle. These fascial layers cause a difference in tension between the puncture sites, with the second puncture site demonstrating higher resistance owing to dense fascial integration (Figure 4). The tension at the second puncture site was approximately twice that at the first puncture site, reflecting the differences in the fascial components of the two sites.

Injection of filler into the deep pyriform space is a well‐established and highly effective technique; however, cases of filler migration have been reported [8, 9, 10, 11, 12, 13]. Although such complications are relatively uncommon, they may compromise aesthetic outcomes and reduce patient satisfaction. Histological analysis in this study confirmed that the nasalis muscle partially forms the lateral boundary between the spaces (Figure 3). The resulting fascial structure provides a clearly defined anatomical separation, effectively creating a stable lateral wall for the perialar space. Anchoring fillers within this well‐demarcated space may reduce the risk of filler migration. Therefore, targeting the perialar space, either alone or in combination with the deep pyriform space, could offer physicians a practical alternative injection site, contributing to enhanced safety and improved aesthetic outcomes in nasolabial fold rejuvenation.

Injection into the perialar space requires traversing the transverse nasalis muscle. A single skin entry point was made using a needle. Through the same entry, the cannula was advanced into the space for the first pass and then, without exiting the skin, it was redirected twice to traverse the fibromuscular barrier, re‐entering the targeted space. This approach allowed for injections at three separate penetration points through a single skin puncture site, ensuring adequate distribution of the filler within the space. When using multiple entry points, the filler can be evenly distributed across the targeted area, ensuring more uniform volumization and better aesthetic outcomes. However, pushing the cannula deeper and with greater force may lead to unintended penetration of the alar fibrofatty tissue, which forms the medial wall of the targeted space. This could result in the inadvertent placement of the filler in the nasal cavity, potentially causing it to extrude.

As shown in Figure 2B, the infraorbital nerve originating from the infraorbital foramen runs superior to the injectate targeting the deep pyriform space. A previous case report suggested that compression caused by filler can lead to sensory loss, which is as important as vascular compromise, granuloma formation, or infection [14]. To reduce this risk and possible vascular penetration, skin perforation lateral to the nasolabial folds, and the guiding of the cannula along the bone surface may reduce the potential for nerve injury [14, 15].

This study has certain limitations. This study was conducted exclusively on Korean cadavers, and anatomical variations may exist among different ethnic groups. Although these findings offer valuable anatomical insights and potential usefulness within this region, caution is advised when generalizing these results to populations with diverse ethnic backgrounds. Additionally, this study did not include a histological analysis of the nasal region. Further research is required to provide histological evidence demonstrating the complete separation of the two spaces. Finally, additional studies are necessary to compare the clinical outcomes of treatments targeting the deep pyriform space versus those targeting the perialar space to evaluate their relative efficacy and patient satisfaction.

In conclusion, this cadaveric study highlights the distinct anatomical boundaries between the deep pyriform and perialar spaces. The penetration force at the second puncture site was significantly higher than that at the first puncture site, indicating the presence of denser fascial layers when penetrating the perialar space than when penetrating the deep pyriform space. This finding suggests that a filler injection into the perialar space may reduce the risk of migration, enhancing the safety and efficacy of nasolabial fold augmentation.

## Author Contributions

**Song Eun Yoon:** conceptualization, validation, formal analysis, investigation, writing – original draft, writing – review and editing, supervision, project administration. **Jung‐Woo Choi:** software, formal analysis, investigation, resources, data curation, writing – original draft, writing – review and editing, visualization, project administration. **Yong‐Seok Nam:** conceptualization, methodology, software, validation, formal analysis, investigation, data curation, writing – original draft, writing – review and editing, supervision, project administration. **In‐Beom Kim:** conceptualization, methodology, formal analysis, resources, writing – review and editing, supervision, project administration.

## Funding

The authors have nothing to report.

## Ethics Statement

Following the Korean Act on the Anatomical Dissection and Preservation of Corpses as amended, this study was performed after review and receiving ethical approval from the Institutional Review Board of the College of Medicine, The Catholic University of Korea (approval number: MC21EISI0103), and all procedures complied with relevant institutional guidelines, ethical standards, and applicable laws governing the use of cadaveric materials for research.

## Consent

Written informed consent was obtained from the body donor themselves when they were alive and from the next of kin when they passed away.

## Conflicts of Interest

The authors declare no conflicts of interest.

## Data Availability

The data that support the findings of this study are available from the corresponding author upon reasonable request.
